# Homothetic symmetries of static plane symmetric spacetimes: A comparative study of energy-momentum tensor representations

**DOI:** 10.1371/journal.pone.0356201

**Published:** 2026-08-28

**Authors:** Wajid Ullah, Fawad Khan, Laila A. Al-Essa

**Affiliations:** 1 Department of Mathematics, University of Peshawar, Khyber Pakhtunkhwa, Pakistan; 2 Department of Mathematical Sciences, College of Science, Princess Nourah bint Abdulrahman University, Riyadh, Saudi Arabia; Yibin University, CHINA

## Abstract

This research explores homothetic matter collineations of static plane symmetric space-times through a comprehensive analysis of the stress-energy tensor’s contravariant, co-variant and mixed representations. When dealing with non-degenerate energy-momentumtensors, the contravariant formulation produces finite-dimensional Lie algebras of dimen-sion 4, 5, 6, and 11, while the mixed tensor form consistently generates infinite-dimensional Lie algebras. For degenerate cases, the solution of homothetic matter collineations equations reveals infinite-dimensional Lie algebras for both tensor representations. Further-more, the present study provides a comparative analysis between the homothetic mattercollineations obtained for contravariant and mixed representations of energy-momentumtensors and those obtained for the covariant formulation in an earlier work, identifying significant distinctions among the three approaches used for studying homothetic matter collineations.

## 1. Introduction

General relativity, widely regarded as one of the definitive theories in modern physics, describes gravity as a manifestation of spacetime curvature determined by matter and energy distributions. This fundamental theory is mathematically expressed through a system of ten nonlinear partial differential equations known as Einstein’s field equations (EFEs) [1].


$$\begin{array}{c} G_{ab}=R_{ab}-\frac{1}{2}R\;g_{ab}=\kappa T_{ab}. \end{array}$$ (1.1)

In this formulation, $G_{ab}$ represents the Einstein tensor encoding spacetime curvature, $R_{ab}$ is the Ricci tensor, $T_{ab}$ describes the matter-energy content, and $g_{ab}$ defines the spacetime metric. The scalar curvature *R* and the coupling constant κ complete the relationship between geometry and physics. Due to their inherent nonlinearity, finding exact solutions to the EFEs is highly challenging, which explains why only a few physically significant exact solutions are known in the literature. Eq. (1.1) shows the covariant version of the EFEs, while the contravariant form is given by:


$$\begin{array}{c} G^{ab}=\kappa T^{ab}. \end{array}$$ (1.2)

These equations may seem simple at first look, but they are, in fact, highly nonlinear and present considerable difficulties when attempting to solve them. Nevertheless, certain exact solutions of these equations with physical relevance have been explored in Refs. [1–3].

The study of exact solutions to the EFEs highly depends on spacetime symmetries, which also help uncover their physical implications. A prime example is spherical symmetry, which validates the Schwarzschild solution and clarifies why a spherically oscillating star emits no gravitational radiation. Spacetime symmetries are governed by vector fields that preserve key features of spacetime including geodesic structure, metric, curvature, or matter distribution.

A vector field *X* on a manifold *M* is called homothetic matter collineations (HMCs) if its action (via the Lie derivative) scales the $T^{ab}$ by a constant factor α:


$$\begin{array}{c} {ℒ}_{X}T^{ab}=2\alpha T^{ab}. \end{array}$$ (1.3)

Here $T^{ab}$ defines stress-energy tensor in its contravariant form. In explicit form, the preceding relation can be expressed as:


$$\begin{array}{c} T^{ab}{,}_{c}X^{c}-T^{ac}X^{b}{,}_{c}-T^{bc}X^{a}{,}_{c}=2\alpha T^{ab}. \end{array}$$ (1.4)

In case of covariant form $T_{ab}$ of the stress-energy tensor, the HMCs are defined as:


$$\begin{array}{c} {ℒ}_{X}T_{ab}=T_{ab,c}X^{c}+T_{ac}X_{,b}^{c}+T_{bc}X_{,a}^{c}=2\alpha T_{ab}. \end{array}$$ (1.5)

where as these collineations for the mixed form $T_{b}^{a}$ of stress-energy tensor are defined as:


$$\begin{array}{c} {ℒ}_{X}T_{b}^{a}=T_{b,c}^{a}X^{c}-T_{b}^{c}X_{,c}^{a}+T_{c}^{a}X_{,b}^{c}=2\alpha T_{b}^{a}. \end{array}$$ (1.6)

In particular, if α=0, each of the equations (1.4)-(1.6) give matter collineations (MCs). For α=0, the energy-momentum tensor (EMT) is exactly preserved, that is the Lie derivative of EMT vanishes. Therefore MCs represent exact symmetries of the matter distribution. Physically, MCs are associated with transformations under which the energy density, pressure, and momentum flux remain invariant. On the other hand, if α≠0, then Eqs. (1.4)-(1.6) give rise to proper HMCs which preserve the EMT only up to a constant scale factor. The proper HMCs describe self-similar matter configurations whose physical properties are not invariant but scale uniformly along the flow generated by the vector field. As a result, scaling properties of the matter content are revealed by proper HMCs, while MCs characterize its exact conservation under symmetry transformations.

The homothetic symmetries of the Ricci tensor (Ricci collineations) and that of the metric tensor, known as homothetic vector fields (HVFs), are respectively defined in a similar way with $T_{ab}$ replaced by $R_{ab}$ and $g_{ab}$ in Eq (1.5). These symmetries are thoroughly investigated for different spacetime geometries in literature.

Shabbir and Amur [4] classified proper HVFs in Bianchi type-I spacetimes and obtained 4, 5, 7, and 11-dimensional homothetic algebras. Hussain et al. [5] examined Bianchi type-IV spacetimes by considering their proper homothetic symmetries and obtained homothetic algebras of dimensions 4 and 5. Hussain and Rahim [6] classified HMCs for Bianchi type-I spacetimes. In the case of non-degenerate stress-energy tensor, Lie algebras with dimensions 6, 7, 8, 10 and 11 were obtained, whereas in degenerate case some sub cases produced 6- and 11-dimensional algebras of HMCs, while the remaining yield HMCs of infinite dimensions. Hussain *et al.* [7] examined HMCs of Bianchi type V spacetimes with a perfect fluid source. When the stress-energy tensor was assumed to be non-degenerate, their study derived four distinct cases, each corresponding to a 5-dimensional Lie algebra. In the degenerate scenario, two possibilities arise, the first case admitted an 11-dimensional Lie algebra, while the second case gave an infinite-dimensional Lie algebra. Hussain *et al.* [8] examined static plane symmetric spacetimes by considering their covariant HMCs and obtained Lie algebra of dimensions 6, 7, 8, 10, and 11, when $T_{ab}$ is taken to be non-degenerate. In degenerate case, two sub cases produced finite-dimensional algebras with dimensions 6 and 11, while the remaining cases yield infinite-dimensional HMCs. Shabbir and Ramzan [9] classified cylindrically symmetric static spacetimes according to their proper HVFs by direct integration technique. The authors obtained homothetic Lie algebras of dimensions 4, 5, 7, and 11. Hall and Steele [10] investigated spacetime homothety groups characterized by an *r*-dimensional Lie algebra of HVFs, requiring at least one proper HVFs. Some other physically important spacetimes are classified via different symmetries in the Refs. [11–21].

As mentioned earlier, the homothetic symmetries of stress-energy tensor can be defined using three different representations of the stress-energy tensor. These three representations do not necessarily give the same results. In 2007, Sharif and Ismaeel [22] conducted a classification of spherically symmetric spacetimes based on their MCs, examining three distinct representations of stress-energy tensor: covariant ($T_{ab}$), contravariant ($T^{ab}$), and mixed ($T_{b}^{a}$) forms. Their analysis revealed that these three classifications are not generally equivalent. Khan et al. [23] extended this work to static plane symmetric spacetimes by investigating MCs for all the three representations of stress-energy tensor. The authors again observed that the three representations produce results that are not equivalent.

In gravitational physics, the HMCs characterize self-similar properties of the matter distribution and provide insight into scaling behaviors of spacetime sources. Due to this feature, these symmetries are of particular interest. Moreover, in the context of studying exact solutions of EFEs, cosmological models, and gravitational collapse, where self-similarity is often helpful in simplifying the field equations, these symmetries play an important role. Therefore, classifying HMCs not only enriches the symmetry analysis of spacetime but also contributes to understanding the possible self-similar structures admitted by different matter configurations.

The present study investigates HMCs in static plane symmetric spacetimes for contravariant ($T^{ab}$) and mixed ($T_{b}^{a}$) representations of the EMT. We also compare their the obtained results with those obtained for covariant representation, presented in [8]. This work is organized as follows: Section 2 establishes the framework for HMCs by analyzing the contravariant form $T^{ab}$ of the EMT. Section 3 explores the corresponding HMCs using the mixed tensor formulation $T_{b}^{a}$ and compares these findings with those derived from the contravariant and covariant approaches. Finally, we conclude with a summary of the key results and their physical implications.

## 2. HMCs for contravariant representation of EMT

The metric of static plane symmetric spacetimes has the form [1]:


$$\begin{array}{c} ds^{2}=e^{f(x)}dt^{2}-dx^{2}-e^{g(x)}[dy^{2}+dz^{2}], \end{array}$$ (2.1)

where *f* and *g* are arbitrary x−dependent functions, and *t* > 0 represents time while *x*,*y* and *z* are the spa*t*ial coordinates and belong to the set of real numbers. The signature of the metric is (+,−,−,−). The minimal symmetry content of this spacetime is characterized by four Killing vectors (KVs):


$$\begin{array}{c} X_{\left(1\right)}=\partial_{t},\;X_{\left(2\right)}=\partial_{y},\;X_{\left(3\right)}=\partial_{z},\;X_{\left(4\right)}=z\partial_{y}-y\partial_{z}. \end{array}$$ (2.2)

The contravariant components of EMT for the above metric are obtained as:


$$\begin{array}{cc} T^{00}(x) & =-\frac{1}{4e^{f(x)}}[3g^{\prime 2}(x)+4g^{\prime \prime }(x)],\\ T^{11}(x) & =\frac{1}{4}[g^{\prime 2}(x)+2f^{\prime }(x)g^{\prime }(x)],\\ T^{22}(x) & =T^{33}(x)=\frac{1}{4e^{g(x)}}[g^{\prime 2}(x)+f^{\prime }(x)g^{\prime }(x)+f^{\prime 2}(x)+2f^{\prime \prime }(x)+2g^{\prime \prime }(x)]. \end{array}$$ (2.3)

The substitution of (2.3) into Eq. (1.4) yields ten HMCs equations:


$$\begin{array}{c} T^{00}{,}_{1}X^{1}-2T^{00}X^{0}{,}_{0}=2\alpha T^{00} \end{array}$$ (2.4)

$$\begin{array}{c} T^{00}X^{1}{,}_{0}+T^{11}X^{0}{,}_{1}=0 \end{array}$$ (2.5)

$$\begin{array}{c} T^{00}X^{2}{,}_{0}+T^{22}X^{0}{,}_{2}=0 \end{array}$$ (2.6)

$$\begin{array}{c} T^{00}X^{3}{,}_{0}+T^{22}X^{0}{,}_{3}=0 \end{array}$$ (2.7)

$$\begin{array}{c} T^{11}{,}_{1}X^{1}-2T^{11}X^{1}{,}_{1}=2\alpha T^{11} \end{array}$$ (2.8)

$$\begin{array}{c} T^{11}X^{2}{,}_{1}+T^{22}X^{1}{,}_{2}=0 \end{array}$$ (2.9)

$$\begin{array}{c} T^{11}X^{3}{,}_{1}+T^{22}X^{1}{,}_{3}=0 \end{array}$$ (2.10)

$$\begin{array}{c} T^{22}{,}_{1}X^{1}-2T^{22}X^{2}{,}_{2}=2\alpha T^{22} \end{array}$$ (2.11)

$$\begin{array}{c} T^{22}(X^{3}{,}_{2}+X^{2}{,}_{3})=0 \end{array}$$ (2.12)

$$\begin{array}{c} T^{22}{,}_{1}X^{1}-2T^{22}X^{3}{,}_{3}=2\alpha T^{22} \end{array}$$ (2.13)

The HMCs for the metric (2.1) are generated by the vector field *X* with components $\left(X^{0},\;X^{1},\;X^{2},\;X^{3}\right)$ appearing in the above equations. In each equation, a comma in the subscript denotes partial derivative with respect to the corresponding spacetime coordinate. In particular, the numbers 0,1,2,3 after commas denote derivatives with respect to *t*,*x*,*y* and *z* respectively. We solve these HMCs equations separa*t*ely for non-degenerate and degenerate cases of the contravariant EMT. This yields the explicit form of HMCs.

Geometrically, $T^{ab}$ acts as a symmetric bilinear form on the spacetime manifold. For $det(T^{ab})\text{≠}0,$ the EMT is referred as non-degenerate and in such a case it has full rank, which impose independent constraints in all the directions of spacetime. The non-degenerate case usually gives finite-dimensional algebras of HMCs. On the other hand, if $det(T^{ab})=0,$ then the EMT is known as degenerate and in such a case it possesses null directions. As a result, the number of independent determining equations is reduced and arbitrary functions are appeared in the symmetry generators, which consequently produce infinite-dimensional algebras of HMCs.

### 2.1. Non-degenerate case

In the scenario where the contravariant stress-energy tensor is non-degenerate, that is $\det(T^{ab})\text{≠}0$, it necessarily follows that none of the components of the contravariant stress-energy tensor can vanish, that is $T^{00}\text{≠}0$, $T^{11}\text{≠}0$, and $T^{22}\text{≠}0$. Integrating the system of Eqs (2.4)-(2.13), we derive a solution to the HMCs equations expressed in terms of unknown functions that depend only on the variables *t* and *x*.


$$\begin{array}{cc} X^{0} & =-\frac{T^{00}}{T^{22}}[\frac{1}{2}(y^{2}+z^{2})G_{t}^{1}+zG_{t}^{2}+yG_{t}^{3}]+G^{4},\\ X^{1} & =-\frac{T^{11}}{T^{22}}[\frac{1}{2}(y^{2}+z^{2})G_{x}^{1}+zG_{x}^{2}+yG_{x}^{3}]+G^{5},\\ X^{2} & =c_{1}[\frac{yz^{2}}{2}-\frac{y^{3}}{6}]+c_{2}yz+c_{3}[\frac{z^{2}}{2}-\frac{y^{2}}{2}]+c_{4}z+yG^{1}+G^{3},\\ X^{3} & =c_{1}[\frac{z^{3}}{6}-\frac{y^{2}z}{2}]+c_{2}[\frac{z^{2}}{2}-\frac{y^{2}}{2}]-c_{3}yz-c_{4}z+zG^{1}+G^{2}, \end{array}$$

where $G^{i}=G^{i}(t,x)$ for *i* = 1,2,3. Eqs. (2.6), (2.7), (2.9), (2.10), (2.12) and the difference of (2.11) and (2.13) are trivially satisfied by the above $X^{i}$s, while the remaining equations give rise to some conditions. In order tointegrability find these integrability conditions, we use the above $X^{i}$s in Eq. (2.4), which yields.


$$\begin{array}{cc} & T_{,1}^{00}\left[-\frac{T^{11}}{T^{22}}\left\{\frac{1}{2}\left(y^{2}+z^{2}\right)G_{x}^{1}(t,x)+zG_{x}^{2}(t,x)+yG_{x}^{3}(t,x)\right\}+G^{5}(t,x)\right]\\ & \;-2T^{00}\left[-\frac{T^{00}}{T^{22}}\left\{\frac{1}{2}\left(y^{2}+z^{2}\right)G_{tt}^{1}(t,x)+zG_{tt}^{2}(t,x)+yG_{tt}^{3}(t,x)\right\}+G_{t}^{4}(t,x)\right]=2\alpha T^{00}. \end{array}$$

Equating the like terms on both sides, we obtain the following integrability conditions.


$$\begin{array}{c} T^{11}\;T^{00}{,}_{1}G_{x}^{1}-2(T^{00}{)}^{2}G_{tt}^{1}=0, \end{array}$$ (2.14)

$$\begin{array}{c} T^{11}\;T^{00}{,}_{1}G_{x}^{2}-2(T^{00}{)}^{2}G_{tt}^{2}=0, \end{array}$$ (2.15)

$$\begin{array}{c} T^{11}\;T^{00}{,}_{1}G_{x}^{3}-2(T^{00}{)}^{2}G_{tt}^{3}=0, \end{array}$$ (2.16)

$$\begin{array}{c} T^{00}{,}_{1}G^{5}-2T^{00}G_{t}^{4}=2\alpha T^{00}. \end{array}$$ (2.17)

Similarly, each of the Eqs (2.5), (2.8) and (2.11) produces four integrability conditions. Consequently, we obtain the following complete list of integrability conditions that further constrain the components of $T^{ab}$.


$$\begin{array}{c} T^{11}\;T^{00}{,}_{1}G_{x}^{i}-2(T^{00}{)}^{2}G_{tt}^{i}=0, \end{array}$$ (2.18)

$$\begin{array}{c} G_{tx}^{i}+\frac{T^{22}}{2T^{00}}(\frac{T^{00}}{T^{22}}{)}_{,1}G_{t}^{i}=0, \end{array}$$ (2.19)

$$\begin{array}{c} (T_{,1}^{11}T^{22}-2T^{11}T_{,1}^{22})G_{x}^{i}+2T^{11}T^{22}G_{xx}^{i}=0, \end{array}$$ (2.20)

$$\begin{array}{c} T^{22}{,}_{1}T^{11}G_{x}^{i}+2k(T^{22}{)}^{2}=0, \end{array}$$ (2.21)

$$\begin{array}{c} T^{00}{,}_{1}G^{5}-2T^{00}G_{t}^{4}=2\alpha T^{00}, \end{array}$$ (2.22)

$$\begin{array}{c} T^{00}G_{t}^{5}+T^{11}G_{x}^{4}=0, \end{array}$$ (2.23)

$$\begin{array}{c} T^{11}{,}_{1}G^{5}-2T^{11}G_{x}^{5}=2\alpha T^{11}, \end{array}$$ (2.24)

$$\begin{array}{c} T^{22}{,}_{1}G^{5}-2T^{22}G^{1}=2\alpha T^{22}. \end{array}$$ (2.25)

Here $G^{i}=G^{i}(t,x)$ for *i* = 1,2,3, and $k=0,c_{2},-c_{3},$ for *i* = 1,2,3 respectively. By solving these constraints, we get an explicit form for the vector field *X* that generates HMCs. One can see that the above integrability conditions are highly nonlinear and cannot be solved easily. In literature, such systems are solved by restricting the components of EMT to satisfy certain conditions. These components depend on the spatial variable *x*, so the most commonly used approach is to discuss the cases where none, one, two or all of these components are constants. Consequently, eight possible cases arise. We solve Eqs. (2.18)-(2.25) for all these cases and conclude that three cases produce maximum number of HMCs, that is 11, out of which four are the minimal KVs, six are MCs and one is a proper HMCs. In two cases, α vanishes and we obtain six and four MCs respectively. In the remaining three cases, we have obtained one proper HMCs and four KVs. The components of the collineation vector *X* for all these cases are presented in Table 1.

**Table 1:** HMCs for Non-Degenerate $T^{ab}$ under the constraints given in second column. Each $c_{i}$ represents a constant that corresponds to HMC.

		
Case	Constraints	HMCs
I	$T_{,1}^{22}=0$, $T_{,1}^{11}=0$, $T_{,1}^{00}\text{≠}0$	$X^{0}=-\frac{1}{x}[z\{-c_{5}\sin t+c_{6}\cos t\}+y\{-c_{7}\sin t+c_{8}\cos t\}]$
		$+\frac{1}{x}\{-c_{9}\sin t+c_{10}\cos t\}+c_{11},$
		$X^{1}=-\alpha x-z\{c_{5}\cos t+c_{6}\sin t\}-y\{c_{7}\cos t+c_{8}\sin t\}+c_{9}\cos t+c_{10}\sin t,$
		$X^{2}=-\alpha y+c_{4}z+x\{c_{7}\cos t+c_{8}\sin t\}+c_{12},$
		$X^{3}=-\alpha z-c_{4}y+x\{c_{5}\cos t+c_{6}\sin t\}+c_{13}$.
II	$T_{,1}^{00}=0$, $T_{,1}^{22}=0,T_{,1}^{11}\text{≠}0$	$X^{0}=-\alpha t-c_{5}z-c_{6}y+c_{7}\int \frac{dx}{\sqrt{T^{11}}}+c_{8},$
		$X^{1}=\sqrt{T^{11}}\left[-c_{9}z-c_{10}y-c_{7}t-\alpha \int \frac{dx}{\sqrt{T^{11}}}\right]+c_{11},$
		$X^{2}=-\alpha y+c_{4}z+c_{5}t+c_{10}\int \frac{dx}{\sqrt{T^{11}}}+c_{12},$
		$X^{3}=-\alpha z-c_{4}y+c_{6}t+c_{9}\int \frac{dx}{\sqrt{T^{11}}}+c_{13}.$
III	$T_{,1}^{00}=0$, $T_{,1}^{11}=0,$ $T_{,1}^{22}\text{≠}0$	$X^{0}=c_{5},\;X^{1}=2z\;c_{2}-2y\;c_{3}+2c_{6},$
		$X^{2}=c_{2}yz+c_{3}\left(\frac{z^{2}}{2}-\frac{y^{2}}{2}\right)+c_{4}z+yc_{6}+2c_{3}e^{x}+c_{7},$
		$X^{3}=c_{2}\left(\frac{z^{2}}{2}-\frac{y^{2}}{2}\right)-c_{3}yz-c_{4}y+zc_{6}-2c_{2}e^{x}+c_{8}$.
IV	$T_{,1}^{00}=0$, $T_{,1}^{11}=0$, $T_{,1}^{22}=0$	$X^{0}=-\alpha t-c_{5}y-c_{6}z+c_{7}x+c_{8},\;X^{1}=-\alpha x-c_{7}t-c_{9}y-c_{10}z+c_{11},$
		$X^{2}=-\alpha y+c_{4}z+c_{5}t+c_{9}x+c_{12}$, $X^{3}=-\alpha z-c_{4}y+c_{6}t+c_{10}x+c_{13}$.
V	$T^{00}=e^{x},$ $T^{11}=e^{2x}$, $T_{,1}^{22}=0$	$X^{0}=-\frac{\alpha t}{2}+c_{5},\;X^{1}=\alpha ,$
		$X^{2}=-\alpha y+c_{4}z+c_{6},\;X^{3}=-\alpha z-c_{4}y+c_{7}$.
VI	$T_{,1}^{00}\text{≠}0,$ $T^{22}=e^{2x}$, $T_{,1}^{11}=0$	$X^{0}=c_{5}$, *X*^1^ = 0,
		$X^{2}=c_{4}z+c_{6},\;X^{3}=-c_{4}y+c_{7}$.
VII	$T^{11}=T^{22}=x,\;T_{,1}^{00}=0$	$X^{0}=-\alpha t+c_{5}$, $X^{1}=-2\alpha x,$
		$X^{2}=-2\alpha y+c_{4}z+c_{6},\;X^{3}=-2\alpha z-c_{4}y+c_{7}$.
VIII	$T_{,1}^{00}\text{≠}0,$ $T_{,1}^{11}\text{≠}0,$ $T_{,1}^{22}\text{≠}0$	$X^{0}=c_{5}$, $X^{1}=2\alpha ,$
		$X^{2}=c_{4}z+c_{6},\;X^{3}=-c_{4}y+c_{7}$.

The static plane symmetric metric (2.1) possesses a minimal isometry group generated by the four Killing Vector Fields (KVFs) mentioned in (2.2). Each of these four KVFs leaves the metric invariant, indicating that all these KVFs represent natural symmetries of the underlying spacetime geometry. These four KVFs are contained as a sub algebra in the algebras of HMCs obtained in the current classification. In some cases, the additional generators are associated with proper MCs (when α=0) or proper HMCs (when α≠0). Thus the obtained algebras of HMCs can be considered as extensions of the isometry algebra, whose dimension depends on the algebraic properties of $T^{ab}$.

In order to see the structure of Lie algebra, we write the generators and compute the corresponding Lie algebra for all the obtained HMCs/MCs and present the results in Tables 2 and 3.

**Table 2:** Lie algebra of the obtained HMCs for Non-Degenerate $T^{ab}$. Each $\upsilon_{i}$ denotes a generator associated with some $c_{i}$ in Table 1.

Case	Generators	Lie Bracket Operation
I	$\upsilon_{1}=\partial_{t},\;\upsilon_{2}=\alpha x\;\partial_{x}+\alpha y\;\partial_{y}+\alpha z\;\partial_{z}$	$[\upsilon_{1},\upsilon_{4}]=\upsilon_{5},\;[\upsilon_{1},\upsilon_{5}]=-\upsilon_{4},$
	$\upsilon_{3}=z\;\partial_{y}-y\;\partial_{z},\;\upsilon_{4}=\frac{z\sin t}{x}\;\partial_{t}+z\cos t\;\partial_{x}-\frac{z\cos t}{x}\;\partial_{y}-\frac{y\sin t}{x}\;\partial_{z}$	$[\upsilon_{2},\upsilon_{8}]=-\upsilon_{8},\;[\upsilon_{2},\upsilon_{9}]=-\upsilon_{9},$
	$\upsilon_{5}=-\frac{z\cos t}{x}\;\partial_{t}+z\sin t\;\partial_{x}-\frac{z\sin t}{x}\;\partial_{y}-\frac{y\cos t}{x}\;\partial_{z}$	$[\upsilon_{3},\upsilon_{5}]=-\upsilon_{7},\;[\upsilon_{3},\upsilon_{6}]=\upsilon_{4},$
	$\upsilon_{6}=\frac{y\sin t}{x}\;\partial_{t}+y\cos t\;\partial_{x}-\cos t\;\partial_{y}-\frac{z\sin t}{x}\;\partial_{z}$	$[\upsilon_{1},\upsilon_{6}]=\upsilon_{7},\;[\upsilon_{1},\upsilon_{7}]=-\upsilon_{6},$
	$\upsilon_{7}=-\frac{y\cos t}{x}\;\partial_{t}+y\sin t\;\partial_{x}-\sin t\;\partial_{y}-\frac{z\cos t}{x}\;\partial_{z}$	$[\upsilon_{1},\upsilon_{8}]=\upsilon_{9},[\upsilon_{1},\upsilon_{9}]=-\upsilon_{8},$
	$\upsilon_{8}=-\frac{\sin t}{x}\;\partial_{t}-\cos t\;\partial_{x}$	$[\upsilon_{2},\upsilon_{10}]=-\alpha \upsilon_{10},\;[\upsilon_{2},\upsilon_{11}]=-\alpha \upsilon_{11},$
	$\upsilon_{9}=\frac{\cos t}{x}\;\partial_{t}-\sin t\;\partial_{x}$	$[\upsilon_{3},\upsilon_{4}]=-\upsilon_{6},[\upsilon_{3},\upsilon_{7}]=\upsilon_{5},$
	$\upsilon_{10}=\partial_{y},\;\;\upsilon_{11}=\partial_{z}\;$	$[\upsilon_{3},\upsilon_{10}]=\upsilon_{11},\;[\upsilon_{3},\upsilon_{11}]=-\upsilon_{10}$.
II	$\upsilon_{1}=\partial_{t},\;\upsilon_{2}=\sqrt{T_{11}}\;\partial_{x},\;\upsilon_{3}=z\;\partial_{y}-y\;\partial_{z}$	$[\upsilon_{1},\upsilon_{4}]=\upsilon_{6},\;[\upsilon_{1},\upsilon_{5}]=-\upsilon_{6}$
	$\upsilon_{4}=-z\;\partial_{t}+t\;\partial_{y}+I\;\partial_{z},\;\upsilon_{5}=-y\;\partial_{t}+t\;\partial_{z}-I\;\partial_{y}$	$[\upsilon_{2},\upsilon_{6}]=-\upsilon_{2},\;[\upsilon_{1},\upsilon_{11}]=-\upsilon_{1}$
	$\upsilon_{6}=I\;\partial_{t}-t\sqrt{T_{11}}\;\partial_{x}+I\;\partial_{y}+I\;\partial_{z},\;\upsilon_{7}=-I\sqrt{T_{11}}\;\partial_{x},$	$[\upsilon_{2},\upsilon_{11}]=-\alpha \upsilon_{2},\;[\upsilon_{3},\upsilon_{4}]=-\upsilon_{5},$
	$\upsilon_{8}=-I\sqrt{T_{11}}\;\partial_{x},\;\upsilon_{9}=\partial_{y},\;\upsilon_{10}=\partial_{z}$	$[\upsilon_{3},\upsilon_{7}]=-\upsilon_{8},\;[\upsilon_{3},\upsilon_{8}]=\upsilon_{7}$
	$\upsilon_{11}=-t\;\partial_{t}-\alpha I\;\partial_{x}-\alpha y\;\partial_{y}-\alpha z\;\partial_{z}$	$[\upsilon_{3},\upsilon_{9}]=\upsilon_{10},\;[\upsilon_{3},\upsilon_{10}]=-\upsilon_{9}$ $[\upsilon_{6},\upsilon_{11}]=-\upsilon_{6},\;[\upsilon_{9},\upsilon_{11}]=-\alpha \upsilon_{9},$ $[\upsilon_{10},\upsilon_{11}]=-\alpha \upsilon_{10},\;[\upsilon_{3},\upsilon_{5}]=\upsilon_{4}$.
III	$\upsilon_{1}=\partial_{t},\;\upsilon_{2}=2z\;\partial_{x}+yz\;\partial_{y}+\left(\frac{z^{2}}{2}-\frac{y^{2}}{2}\right)\partial_{z}-2e^{x}\;\partial_{z},$	$[X_{2},X_{4}]=X_{6},\;[X_{2},X_{5}]=-X_{2},$
	$\upsilon_{3}=-2y\;\partial_{x}+\left(\frac{z^{2}}{2}-\frac{y^{2}}{2}\right)\partial_{y}-yz\;\partial_{z}+2e^{x}\;\partial_{y}$	$[X_{2},X_{6}]=-X_{3},\;[X_{3},X_{4}]=X_{7},$
	$\upsilon_{4}=z\;\partial_{y}-y\;\partial_{z}$	$[X_{3},X_{5}]=-X_{3},\;[X_{3},X_{7}]=-X_{2},$
	$\upsilon_{5}=2\;\partial_{x}+y\;\partial_{y}+z\;\partial_{z},\;\upsilon_{6}=\partial_{y}$	$[X_{4},X_{6}]=X_{7},\;[X_{4},X_{7}]=-X_{6},$
	$\upsilon_{7}=\partial_{z}$	$[X_{5},X_{6}]=-X_{6},\;[X_{5},X_{7}]=-X_{7}$.

**Table 3:** Lie algebra of the obtained HMCs for Non-Degenerate $T^{ab}$. Each $\upsilon_{i}$ denotes a generator associated with some $c_{i}$ in Table 1.

Case	Generators	Lie Bracket Operation
IV	$\upsilon_{1}=\partial_{t},\;\upsilon_{2}=\partial_{x},\;\upsilon_{3}=\partial_{y},\;\upsilon_{4}=\partial_{z}$	$[\upsilon_{1},\upsilon_{6}]=\upsilon_{1},\;[\upsilon_{1},\upsilon_{7}]=\upsilon_{1},$
	$\upsilon_{5}=z\;\partial_{y}-y\;\partial_{z},\;\upsilon_{6}=-y\;\partial_{t}+t\;\partial_{y}+x\;\partial_{z}$	$[\upsilon_{1},\upsilon_{8}]=\upsilon_{2},\;[\upsilon_{1},\upsilon_{11}]=-\upsilon_{1},$
	$\upsilon_{7}=-z\;\partial_{t}+t\;\partial_{z}-x\;\partial_{y},\;\upsilon_{8}=x\;\partial_{t}+t\;\partial_{x},$	$[\upsilon_{2},\upsilon_{8}]=\upsilon_{1},\;[\upsilon_{2},\upsilon_{9}]=-\upsilon_{3},$
	$\upsilon_{9}=-y\;\partial_{x}+x\;\partial_{y},\;\upsilon_{10}=-z\;\partial_{x}+x\;\partial_{z},$	$\;[\upsilon_{2},\upsilon_{10}]=-\upsilon_{4},\;[\upsilon_{2},\upsilon_{11}]=-\upsilon_{2},$
	$\upsilon_{11}=-t\;\partial_{t}-x\;\partial_{x}-y\;\partial_{y}-z\;\partial_{z}$.	$[\upsilon_{3},\upsilon_{4}]=\upsilon_{5},\;[\upsilon_{3},\upsilon_{5}]=-\upsilon_{4},$
		$[\upsilon_{3},\upsilon_{6}]=\upsilon_{4},\;[\upsilon_{3},\upsilon_{7}]=-\upsilon_{2}$.
		$[\upsilon_{3},\upsilon_{9}]=-\upsilon_{1},\;[\upsilon_{3},\upsilon_{11}]=-\upsilon_{3},$
		$[\upsilon_{4},\upsilon_{5}]=\upsilon_{3},\;[\upsilon_{4},\upsilon_{6}]=-\upsilon_{3},$
		$[\upsilon_{4},\upsilon_{7}]=[\upsilon_{4},\upsilon_{10}]=-\upsilon_{1}$
		$[\upsilon_{4},\upsilon_{11}]=-\upsilon_{4},\;[\upsilon_{5},\upsilon_{6}]=\upsilon_{7},$
		$[\upsilon_{5},\upsilon_{7}]=-\upsilon_{6},\;[\upsilon_{5},\upsilon_{9}]=\upsilon_{10},$
		$[\upsilon_{5},\upsilon_{10}]=-\upsilon_{9},\;[\upsilon_{7},\upsilon_{11}]=-\upsilon_{7},$
		$[\upsilon_{8},\upsilon_{11}]=-\upsilon_{8},\;[\upsilon_{9},\upsilon_{10}]=\upsilon_{5}$
		$[\upsilon_{9},\upsilon_{11}]=-\upsilon_{9},\;[\upsilon_{10},\upsilon_{11}]=-\upsilon_{10}$.
V	$\upsilon_{1}=\partial_{t},\;\upsilon_{2}=\partial_{x},$	$[\upsilon_{1},\upsilon_{6}]=-\frac{1}{2}\upsilon_{1},\;[\upsilon_{3},\upsilon_{4}]=\upsilon_{5},$
	$\upsilon_{3}=z\;\partial_{y}-y\;\partial_{z},\;\upsilon_{4}=\partial_{y},\;$	$[\upsilon_{3},\upsilon_{5}]=-\upsilon_{4},\;[\upsilon_{3},\upsilon_{6}]=-\upsilon_{3},$
	$\upsilon_{5}=\partial_{z},\;\upsilon_{6}=-\frac{t}{2}\;\partial_{t}+\partial_{x}-y\;\partial_{y}-z\;\partial_{z}$	$[\upsilon_{4},\upsilon_{6}]=-\upsilon_{4},[\upsilon_{5},\upsilon_{6}]=-\upsilon_{5}$.
VI	$\upsilon_{1}=\partial_{t},\;\upsilon_{2}=\partial_{x},\;\upsilon_{3}=z\;\partial_{y}-y\;\partial_{z}$	$[\upsilon_{3},\upsilon_{4}]=\upsilon_{5},$
	$\upsilon_{4}=\partial_{y},\;\upsilon_{5}=\partial_{z}$	$[\upsilon_{3},\upsilon_{5}]=-\upsilon_{4}$
VII	$\upsilon_{1}=\partial_{t},\;\upsilon_{2}=-t\;\partial_{t}-2x\;\partial_{x}-2y\;\partial_{y}-2z\;\partial_{z}$ $\upsilon_{3}=z\;\partial_{y}-y\;\partial_{z},\;\upsilon_{4}=\partial_{y},\;\upsilon_{5}=\partial_{z}$	$[\upsilon_{1},\upsilon_{2}]=-\upsilon_{1},\;[\upsilon_{2},\upsilon_{3}]=-2\upsilon_{3}$
	$\upsilon_{3}=z\;\partial_{y}-y\;\partial_{z},\;\upsilon_{4}=\partial_{y},\;\upsilon_{5}=\partial_{z}$	$[\upsilon_{2},\upsilon_{4}]=-2\upsilon_{4},\;[\upsilon_{2},\upsilon_{5}]=-2\upsilon_{5},$
		$[\upsilon_{3},\upsilon_{4}]=\upsilon_{5},\;[\upsilon_{3},\upsilon_{5}]=-\upsilon_{4}$.
VIII	$\upsilon_{1}=\partial_{t},\;\upsilon_{2}=\partial_{x},\;\upsilon_{3}=z\;\partial_{y}-y\;\partial_{z}$ $\upsilon_{4}=\partial_{y},\;\upsilon_{5}=\partial_{z}$	$[\upsilon_{3},\upsilon_{4}]=\upsilon_{5},\;[\upsilon_{3},\upsilon_{5}]=-\upsilon_{4}$

### 2.2. Degenerate case

The degeneracy condition of the contravariant stress-energy tensor, that is $det\;T^{ab}=T^{00}T^{11}T^{22}=0,$ implies that one or two components of $T^{ab}$ must vanish, leading to six different possibilities. Solving the HMCs Eqs. (2.4)-(2.13) for all these six cases, we find that the components $X^{i}$s of the collineation vector *X* contain arbitrary functions of the spacetime coordinates. This functional freedom demonstrates that the algebra of HMCs is infinite-dimensional in each of these six cases. The complete set of solutions for all these cases is presented in Table 4.

**Table 4:** HMCs for Degenerate $T^{ab}$ under the constraints given in second column. The appearance of arbitrary functions indicates infinite-dimensionality of algebra of HMCs.

Case	Constraints	HMCs
I	$T^{00}=T^{11}=0$, $T^{22}\text{≠}0$	$X^{0}=G^{1},\;X^{1}=G^{2}$, $X^{2}=-\alpha y+\frac{y}{2}\frac{T_{,1}^{22}}{T^{22}}G^{2}+zG^{3}+G^{4}$, $X^{3}=-\alpha z-yG^{3}+\frac{T_{,1}^{2}}{2T^{22}}zG^{2}+G^{5},$ where $G^{i}$s are functions of *t* and *x*.
II	$T^{11}=T^{22}=0$, $T^{00}\text{≠}0$	$X^{0}=-\alpha t+\frac{T_{,1}^{00}}{T^{00}}F^{1}+F^{4}$, $X^{1}=F^{1},\;X^{2}=F^{2},\;X^{3}=F^{3},$ where each $F^{i}$ depends on *x*, *y* and *z*.
III	$T^{00}=T^{22}=0$, $T^{11}\text{≠}0$	$X^{0}=F^{1}$ $X^{1}=\alpha \sqrt{T^{11}}\int \frac{dx}{\sqrt{T^{11}}}+\sqrt{T^{11}}yH^{2}(t)+H^{3}(t)+zH^{4}(t)+H^{5}(t)$, $X^{2}=F^{2}$, $X^{3}=F^{3}$. where $F^{i}$s depend on *t*, *y* and *z*.
IV	$T^{00}\text{≠}0$, $T^{11}\text{≠}0,$ *T*^22^ = 0	$X^{0}=-yH^{1}(z)\int \frac{dx}{{\sqrt{T}}^{11}}+G^{6}(t,z)\int \frac{dx}{{\sqrt{T}}^{11}}+G^{6}(t,z)+G^{5}(y,z),$ $X^{1}=\alpha \sqrt{T^{11}}\int \frac{dx}{\sqrt{T^{11}}}+\sqrt{T^{11}}[yG^{3}(t,z)+G^{4}(t,z)+G^{5}(y,z),$ $X^{2}=G^{1}(y,z),\;X^{3}=G^{2}(y,z)$.
V	$T^{00}\text{≠}0$, $T^{22}\text{≠}0,$ *T*^11^ = 0	$X^{0}=-\alpha t+\frac{T_{,1}^{00}}{2T^{00}}tH^{1}(x)+yH^{2}(x)+G^{3}(x,z)$, $X^{1}=H^{1}(x),$ $X^{2}=-\alpha y-\frac{T_{,1}^{22}}{2T^{22}}tH^{1}(x)-\frac{T^{22}}{T^{00}}tH^{2}(x)+yH^{3}(x)+H^{5}(x),$ $X^{3}=-\alpha z+\frac{T_{,1}^{22}}{2T^{22}}zH^{1}(x)-\frac{T^{22}}{T^{00}}[tyH^{2}(x)-G_{z}^{4}(x,z)+yH^{3}(x)+H^{4}(x)]$.
VI	$T^{11}\text{≠}0$, $T^{22}\text{≠}0,$ *T*^00^ = 0	$X^{0}=G^{1}$, $X^{1}=G^{2}$, $X^{2}=-\alpha y+\frac{T_{,1}^{22}}{2T^{22}}yG^{2}+zG^{3}+G^{4}$, $X^{3}=-\alpha z+\frac{T_{,1}^{22}}{2T^{22}}zG^{2}-yG^{3}+G^{5},$ where $G^{i}$s are functions of *t*, *y*.

Due to the vanishing of one or more components of the EMT in degenerate case, the rank of the determining system for the symmetry equations is reduced, which results disappearance of some differential constraints while some components of the vector field defining HMCs depend on arbitrary functions of the spacetime coordinates. The appearance of arbitrary functions in the components of the HMCs vector field represents genuine degrees of freedom arising from the reduced system rather than gauge or coordinate artifacts. In such cases, the solution space involves several linearly independent symmetry generators, leading to an infinite-dimensional algebra of HMCs.

## 3. HMCs for Mixed Representation of EMT

This section presents sHMC for the metric (2.1) for the mixed form of EMT. The definition of HMCs for mixed representation of EMT is already given in Eq.(1.6), while the components of EMT $T_{b}^{a}$ can be obtained from those of $T^{ab}$ through the contraction, $T_{b}^{a}=g_{bc}T^{ac},$ which explicitly gives:


$$\begin{array}{cc} T_{0}^{0} & =\frac{1}{4}[3g^{\prime 2}(x)+4g^{\prime \prime }(x)],\\ T_{1}^{1} & =-\frac{1}{4}[g^{\prime 2}(x)+2f^{\prime }(x)g^{\prime }(x)],\\ T_{2}^{2} & =T_{3}^{3}=-\frac{1}{4}[g^{\prime 2}(x)+f^{\prime }(x)g^{\prime }(x)+f^{\prime 2}(x)+2f^{\prime \prime }(x)+2g^{\prime \prime }(x)]. \end{array}$$ (3.1)

Using these components of $T_{b}^{a}$ in Eq. (1.6), we obtain the following set of HMCs equations:


$$\begin{array}{c} T_{0,1}^{0}X^{1}=2\alpha T_{0}^{0} \end{array}$$ (3.2)

$$\begin{array}{c} (T_{0}^{0}-T_{1}^{1})X^{0}{,}_{1}=0 \end{array}$$ (3.3)

$$\begin{array}{c} (T_{0}^{0}-T_{1}^{1})X^{1}{,}_{0}=0 \end{array}$$ (3.4)

$$\begin{array}{c} (T_{0}^{0}-T_{2}^{2})X^{0}{,}_{2}=0 \end{array}$$ (3.5)

$$\begin{array}{c} (T_{0}^{0}-T_{2}^{2})X^{2}{,}_{0}=0 \end{array}$$ (3.6)

$$\begin{array}{c} (T_{0}^{0}-T_{2}^{2})X^{0}{,}_{3}=0 \end{array}$$ (3.7)

$$\begin{array}{c} (T_{0}^{0}-T_{2}^{2})X^{3}{,}_{0}=0 \end{array}$$ (3.8)

$$\begin{array}{c} T_{1,1}^{1}X^{1}=2\alpha T_{1}^{1} \end{array}$$ (3.9)

$$\begin{array}{c} (T_{1}^{1}-T_{2}^{2})X^{1}{,}_{2}=0 \end{array}$$ (3.10)

$$\begin{array}{c} (T_{1}^{1}-T_{2}^{2})X^{2}{,}_{1}=0 \end{array}$$ (3.11)

$$\begin{array}{c} (T_{1}^{1}-T_{2}^{2})X^{1}{,}_{3}=0 \end{array}$$ (3.12)

$$\begin{array}{c} (T_{1}^{1}-T_{2}^{2})X^{3}{,}_{1}=0 \end{array}$$ (3.13)

$$\begin{array}{c} T_{2,1}^{2}X^{1}=2\alpha T_{2}^{2} \end{array}$$ (3.14)

As compared to the covariant and contravariant forms, a fundamentally different structure of the HMCs equations is obtained by the mixed representation of EMT. We can see that the symmetry Eqs (3.2)-(3.14) involve many equations containing the differences of diagonal components of EMT. As these components only depend on the spatial variable *x*, the resulting symmetry equations do not impose any restrictions on the derivatives of the symmetry vector field with respect to *t*,*y*, and *z*. Hence arbitrary functional dependencies are produced in the components of *X*, that yield enlarge solution space and we obtain infinite-dimensional Lie algebra of symmetry vector fields.

To facilitate a comprehensive comparison among the HMCs obtained for different representations of EMT, we have solved the above equations for the degenerate and non-degenerate cases, same as in the previous section. Remarkably, here all cases of degenerate as well as non-degenerate cases yield infinite-dimensional HMCs, indicating a richer symmetry structure than that obtained in the previous section.

## 4. Comparison of different representations of EMT

Though the three different representations of EMT are related by index raising and lowering, the symmetry equations for all the three cases are generally not equivalent. The reason behind this feature is that the operations of raising and lowering involve the modification of symmetry equations through the metric. As a result, different symmetry conditions and the corresponding Lie algebras may arise. Generally, there is no specific condition under which the contravariant, covariant and mixed representations coincide. Moreover, weaker constraints are admitted by the mixed representation of EMT, leading to arbitrary functions in the components of the vector field and infinite-dimensional algebras of HMCs.

In the previous two sections, we have solved the HMCs equations for static plane symmetric spacetime by considering the EMT in its contravariant and mixed forms. As a result, the degenerate EMT for both contravariant and mixed forms produced infinite number of HMCs. For mixed form, the non-degenerate EMT also yields infinite HMCs. However in case of contravariant form of EMT, some of the non-degenerate cases give five and eleven HMCs, while the other cases produce four and six MCs with no proper HMCs.

The HMCs for the same spacetime with covariant representation of EMT were explored in Ref. [8]. For two sub cases of the degenerate EMT in Ref. [8], the authors obtained finite-dimensional algebra of HMCs with dimension 6 and 11. However the same degenerate case of the present study of HMCs produced infinite set of HMCs for all it sub cases. Moreover, the non-degenerate EMT in Ref. [8] produced 6-, 7-, 8-, 10- and 11-dimensional algebra of HMCs for different sub cases, while the present study yielded 4, 5, 6 and 11 HMCs for the same non-degenerate EMT in its contravariant form. Table 5 compares our derived results for contravariant ($T^{ab}$) and mixed ($T_{b}^{a}$) EMT formulations with those given in Ref. [8], which employed the covariant form $T_{ab}$ of EMT.

**Table 5:** Comparison of HMCs obtained from different representations of EMT.

Case No.	${ℒ}_{X}T_{ab}=2\alpha T_{ab}$	${ℒ}_{X}T^{ab}=2\alpha T^{ab}$	${ℒ}_{X}T_{b}^{a}=2\alpha T_{b}^{a}$
I	6 (MCs)	11 (HMCs)	Infinite Dimensional
II	11 (HMCs)	11 (HMCs)	Infinite Dimensional
III	7 (MCs)	6 (MCs)	Infinite Dimensional
IV	11 (HMCs)	11 (HMCs)	Infinite Dimensional
V	11 (HMCs)	5 (HMCs)	Infinite Dimensional
VI	8 (HMCs), 10 (MCs)	4 (MCs)	Infinite Dimensional
VII	7 (MCs)	5 (HMCs)	Infinite Dimensional
VIII	8 (HMCs), 10 (MCs)	5 (HMCs)	Infinite Dimensional

## 5 Some specific metrics satisfying the desired EMT conditions

We can see that in some cases, the obtained HMCs in the current study are subject to specific conditions on EMT components. In this section, we provide some specific examples of metric functions satisfying these conditions.

For *f*(*x*) = 0 and *g*(*x*) = *x*, the contravariant components of EMT turn out to be $T^{00}=-\frac{3}{4},T^{11}=\frac{1}{4}$ and $T^{22}=T^{33}=\frac{1}{4e^{x}}$. These components satisfy the conditions of case III presented in Table 1.

Another example can be constructed using *f*(*x*) = *x* + 1 and *g*(*x*) = 2(*x* + 1). For these functions, the components of $T^{ab}$ are found to be $T^{00}=-\frac{3}{e^{x+1}},T^{11}=2$ and $T^{22}=T^{33}=\frac{7}{4e^{2(x+1)}}$. These terms clearly satisfy the constraints of case VI in Table 1.

Similarly, for *f*(*x*) = *x*^2^ and *g*(*x*) = *x*, the EMT components take the form $T^{00}=\frac{1}{4e^{x^{2}}},T^{11}=\frac{4x+1}{4}$ and $T^{22}=T^{33}=\frac{4x^{2}+2x+4}{4e^{x}},$ that clearly satisfy the condition of case VIII in Table 1.

In case of degenerate EMT, similar type of specific metrics can be constructed satisfying the required conditions. For example, if *f*(*x*) = *x* and *g*(*x*) = 0, then $T^{00}=T^{11}=0$ and $T^{22}=T^{33}=\frac{3}{4e^{x^{2}}},$ that satisfy the constraints of case I of degenerate case presented in table 4. Similarly, the functions $f(x)=\frac{1}{2x}$ and $g(x)=\ln x^{2}$ lead to $T^{00}=-\frac{1}{4e^{\frac{1}{2x}}}(\frac{3}{x^{4}}-\frac{8}{x^{3}})$ *T*^11^ = 0 and $T^{22}=T^{33}=\frac{1}{4x^{2}}(\frac{3}{4x^{4}}-\frac{2}{x^{3}}),$ which satisfy the conditions of case IV of degenerate case presented in table 4.

## 6 Conclusion

We have explored HMCs for the metric (2.1) focusing on the contravariant and mixed forms of the stress-energy tensor. Previous work in Ref. [8] analyzed HMCs of the same metric for covariant form of the stress-energy tensor. By solving the HMCs equations for both degenerate and non-degenerate cases, we observe distinct patterns in the Lie algebra dimensions.

For the degenerate stress-energy tensor in contravariant and mixed forms, the HMCs become infinite-dimensional due to the presence of arbitrary functions in the vector field components. Interestingly, Ref. [8] reported both finite and infinite-dimensional HMCs for the covariant form under degenerate conditions, highlighting a contrast in the results.

For the non-degenerate contravariant stress-energy tensor, our analysis reveals a finite-dimensional Lie algebra with dimensions 5 and 11 (HMCs), and 4 and 6 (MCs). However, the covariant case studied in Ref. [8] yielded a broader range of dimensions, including 6, 7, 8, 10, and 11. Additionally, while examining the degenerate mixed form we find an infinite-dimensional Lie algebra, whereas the covariant form in the referenced work produced finite dimensions of 6 and 11.

In cases where the integrability conditions impose minimal restrictions on the unknown functions defining the HVFs, we have obtained the homothetic algebra of maximal dimension. In such cases, additional functional constraints are satisfied by the EMT, such as vanishing or constant derivatives. This feature converts the symmetry equations to a maximally integrable form, and the solution space possesses the complete set of independent generators containing four KVFs, six MCs that arise from residual spatial and scaling freedoms, and one proper HMCs. Consequently, maximal 11-dimensional Lie algebra is produced.

The comparative analysis of all the three froms of EMT highlights that the classification of HMCs is highly sensitive to the chosen representation of the EMT. In particular, while the contravariant and covariant forms admit finite-dimensional algebras in several non-degenerate cases, the mixed representation invariably yields infinite-dimensional structures, revealing a richer but less restrictive symmetry content. Such distinctions are not merely formal; they suggest that physical predictions involving spacetime symmetries, ranging from compact star models to anisotropic cosmologies, may depend on the adopted EMT formulation. This observation opens new perspectives for exploring generalized matter models and even extensions of general relativity, where the role of different EMT representations remains largely unexplored.

In Refs. [24–27], the authors have explored ricci collineations (RCs), curvature collineations (CCs), HVFs and conformal vector fields (CVFs) for the same static plane symmetric spacetime. In case of RCs, the authors obtained 4, 5, 6, 7 and 10 RCs for non-degenerate Ricci tensor, while infinitely many RCs were obtained for degenerate case [24]. The same number of CCs was obtained in Ref. [25]. In the study of HVFs, the authors obtained one proper homothety along with 4, 5, 6, 7 and 10 KVs [26]. In Ref. [27], the authors derived some plane symmetric metrics possessing one proper CVFs along with one proper homothety and four KVFs. In the preset analysis, we have also obtained the same number of MCs with one extra HMCs in some cases for contravariant representation of EMT, which is same as that of covariant representation. However, the case of degenerate EMT is similar to that of the degenerate case of the study of RCs.

Beyond the mathematical classification of symmetries, the obtained results in the present work also have physical significance. For several contravariant cases, the algebra of HMCs was found to be finite-dimensional, which indicate relatively constrained self-similar matter distributions. In contrast, the mixed representation of EMT gives rise to infinite-dimensional algebras which reflect a much richer symmetry structure associated with weaker constraints on the EMT. Consequently, the current classification yields a useful framework for exploring self-similar matter configurations, comparing different representations of the EMT, and supporting future studies of exact solutions, anisotropic cosmological models, and other gravitational systems where matter symmetries play a fundamental role.
